# Competitive endogenous RNA (ceRNA) regulation network of lncRNAs, miRNAs, and mRNAs in Wilms tumour

**DOI:** 10.1186/s12920-019-0644-y

**Published:** 2019-12-16

**Authors:** Fucai Tang, Zechao Lu, Jiamin Wang, Zhibiao Li, Weijia Wu, Haifeng Duan, Zhaohui He

**Affiliations:** 1 Department of Urology, The Eighth Affiliated Hospital, Sun Yat-sen University, Shenzhen, 518033 China; 2 First Clinical College of Guangzhou Medical University, Guangzhou, 510230 China; 3 Department of Urology, Minimally Invasive Surgery Center, Guangdong Provincial Key Laboratory of Urology, The First Affiliated Hospital of Guangzhou Medical University, Guangzhou, 510230 China; 4 Three Clinical College of Guangzhou Medical University, Guangzhou, 510230 China

**Keywords:** Wilms tumour, Long noncoding RNA, Prognosis, Competing endogenous RNA network, MicroRNAs

## Abstract

**Background:**

Competitive endogenous RNAs (ceRNAs) have revealed a new mechanism of interaction between RNAs. However, an understanding of the ceRNA regulatory network in Wilms tumour (WT) remains limited.

**Methods:**

The expression profiles of mRNAs, miRNAs and lncRNAs in Wilms tumour samples and normal samples were obtained from the Therapeutically Applicable Research to Generate Effective Treatment (TARGET) database. The EdgeR package was employed to identify differentially expressed lncRNAs, miRNAs and mRNAs. Functional enrichment analyses via the ClusterProfile R package were performed, and the lncRNA–miRNA–mRNA interaction ceRNA network was established in Cytoscape. Subsequently, the correlation between the ceRNA network and overall survival was analysed.

**Results:**

A total of 2037 lncRNAs, 154 miRNAs and 3609 mRNAs were identified as differentially expressed RNAs in Wilms tumour. Of those, 205 lncRNAs, 26 miRNAs and 143 mRNAs were included in the ceRNA regulatory network. The results of Gene Ontology (GO) analysis revealed that the differentially expressed genes (DEGs) were mainly enriched in terms related to response to mechanical stimuli, transcription factor complexes, and transcription factor activity (related to RNA polymerase II proximal promoter sequence-specific DNA binding). The results of the Kyoto Encyclopedia of Genes and Genomes (KEGG) pathway analysis showed that the DEGs were mainly enriched in pathways related to the cell cycle. The survival analysis results showed that 16 out of the 205 lncRNAs, 1 out of 26 miRNAs and 5 out of 143 mRNAs were associated with overall survival in Wilms tumour patients (*P* < 0.05).

**Conclusions:**

CeRNA networks play an important role in Wilms tumour. This finding might provide effective, novel insights for further understanding the mechanisms underlying Wilms tumour.

## Background

Wilms tumour (WT) is the most common renal malignant tumour in children. WT patients have a poor prognosis, although the 5-year overall survival rate is constantly improving with the advancement of disease-associated therapies [1]. Chemotherapy, surgery and radiation therapy are the main treatment strategies for WT. However, 50% of children who have a recurrence after these treatments die from this tumour [2, 3]. Novel therapeutic treatments targeting specific mechanisms of WT are still lacking.

Previous studies have demonstrated that numerous key long non-coding RNAs (lncRNAs), microRNAs (miRNAs) and mRNAs are closely related to the pathogenesis of WT, such as LINC00473 [4], miR-483-5p [5], miR-195 [4] and HACE1 [6]. However, there are few reports on the development of prognostic biomarkers in WT. If WT patients who were more likely to have a poor prognosis according to these prognosis biomarker results could be identified, clinicians might be able to apply more aggressive and individualized treatment. Therefore, prognostic biomarkers and targeted therapies in WT need to be identified to improve the clinical outcomes.

In the last decade, the complexity of the human genome has been revealed by advanced RNA sequencing analysis [7]. Under such circumstances, competing endogenous RNA (ceRNA) analyses have demonstrated that lncRNAs can communicate with common miRNA response elements and miRNAs to construct an intricate interconnected network and ultimately crosstalk with mRNA. The involvement of the ceRNA regulatory network in tumour initiation and progression has been validated in previous studies [8, 9]. However, the specific ceRNA regulatory network (lncRNA–miRNA–mRNA) in WT remains to be elucidated.

In the present study, a ceRNA regulatory network (205 mRNAs, 26 lncRNAs and 143 miRNAs) was constructed to promote the understanding of how lncRNAs sponge miRNA to regulate gene expression in WT. Subsequently, survival analysis and functional analysis were used to promote a new understanding of the role of the ceRNA regulatory network in WT carcinogenesis. The present study might provide insight into the molecular mechanisms that participate in the progression and tumorigenesis of WT.

## Methods

### Data collection and preprocessing

**Fig. 1:**
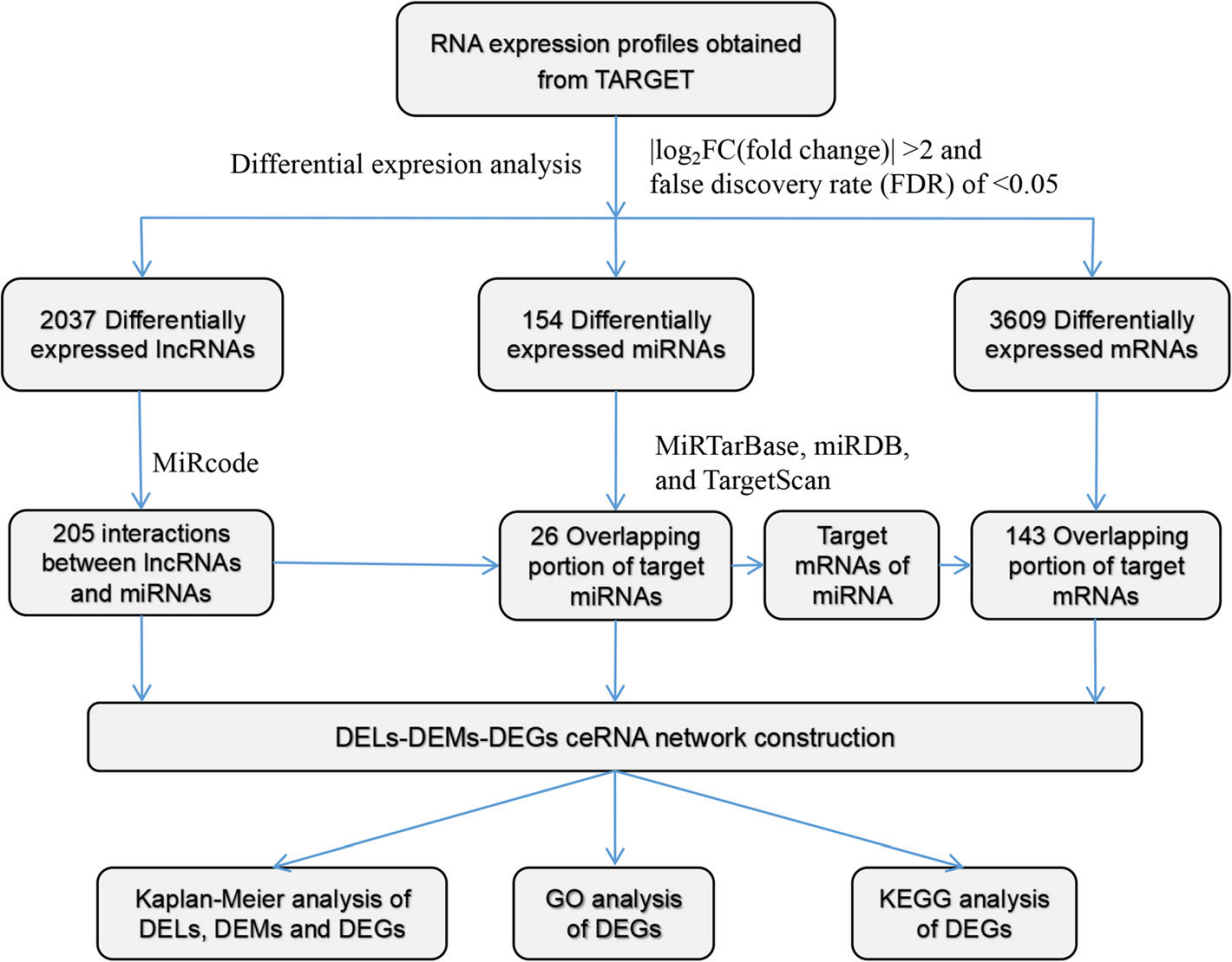
The flow chart of the analysis procedure. TARGET: Therapeutically Applicable Research to Generate Effective Treatment; DELs: differentially expressed lncRNAs; DEMs: differentially expressed miRNAs; DEGs: differentially expressed genes; GO: Gene Ontology; KEGG: Kyoto Encyclopedia of Genes and Genomes

The raw expression data and corresponding clinical follow-up information were retrieved from the Therapeutically Applicable Research to Generate Effective Treatment (TARGET: https://ocg.cancer.gov/programs/target; date: February 2019) database. The data (of lncRNAs, mRNAs and miRNAs) in the present study are publicly available. Ethics committee approval was not required because the data in the present study were obtained from the TARGET database. Among the data, miRNA expression data were acquired from 138 samples, including 6 normal samples and 132 WT samples. In addition, mRNA and lncRNA expression data were acquired from 132 samples, including 6 normal samples and 126 WT samples. The differentially expressed lncRNAs (DELs), differentially expressed miRNAs (DEMs) and differentially expressed genes (DEGs) between WT and normal samples were determined via the EdgeR package in the R software (version 3.3.2) [10]. For the cut-off criteria, a |log_2_FC (fold change)| >2 and a false discovery rate (FDR) of <0.05 were used. A flow chart of the analysis procedure is shown in Fig. 1.

### Prediction of lncRNA–miRNA and miRNA–mRNA interactions

DEL-DEM-DEG interactions were divided into DEL-DEM and DEM-DEG pairs. The StarBase database (*http://starbase.sysu.edu.cn/*) was employed to change the miRNA sequences. MiRcode [11] (http://www.mircode.org/) is an effective online software that provides the interactions between lncRNAs and miRNAs. MiRTarBase [12] (http://mirtarbase.mbc.nctu.edu.tw), miRDB [13] (http://www.mirdb.org/miRDB/), and TargetScan [14] (http://www.targetscan.org/) are online tools that were used to retrieve and predict target mRNAs of the miRNAs. A Venn diagram was constructed to obtain the overlapping portion of target miRNAs and mRNAs. Matched DEL-DEM and DEM-DEG interactions were screened for further bioinformatics analysis.

### CeRNA network construction and functional enrichment analysis

Cytoscape software (Version 3.6.1) was utilized to construct and visualize the DEL-DEM-DEG ceRNA network. Cytoscape was used to visualize the molecular interaction networks according to gene expression profiles and annotations. To better comprehend the tumorigenesis mechanisms in WT, Gene Ontology (GO) functional enrichment analysis and Kyoto Encyclopedia of Genes and Genomes (KEGG) pathway enrichment analysis of DEGs in the ceRNA network were performed via the ClusterProfile R package. An FDR < 0.05 was used as a cut-off value.

### Survival analysis

Identification of prognostic DEL, DEM and DEG signatures were executed via the log-rank test and Kaplan-Meier analysis. The survival curves were constructed using the ‘survival’ package. Survival analysis was performed according to Kaplan-Meier univariate survival analysis. *P* < 0.05 was selected as the statistical threshold value. All survival analyses were conducted using R software (version: 3.3.2).

## Results

### DEL, DEM and DEG identification in WT

**Fig. 2:**
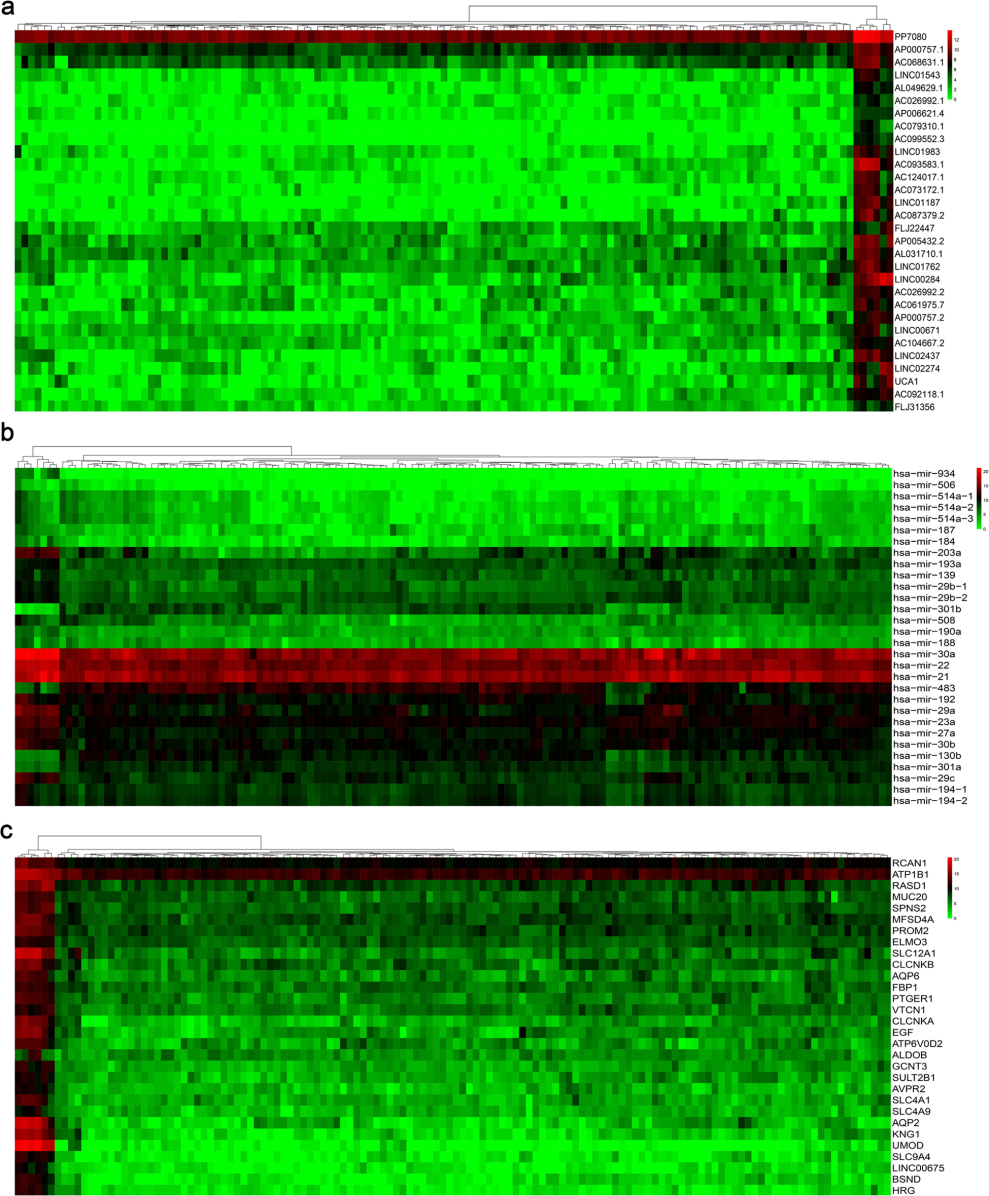
Heatmap analysis of differentially expressed lncRNAs (**a**), miRNAs (**b**), and mRNAs (**c**) (top 30). Each row represents a sample, and each column represents an lncRNA, miRNA, or mRNA. High or low relative expression is displayed as a red or green strip, respectively

LncRNA, mRNA, and miRNA expression profiles between WT samples and normal samples acquired from TARGET were analysed in the present study. A total of 2037 DELs, 154 DEMs and 3609 DEGs were identified in the present study. A total of 1247 upregulated and 790 downregulated DELs were identified in WT with a cut-off threshold of a |log_2_FC (fold change)| > 2 and a false discovery rate (FDR) of < 0.05. The distribution of DELs between WT and normal controls is presented as a heatmap plot in Fig. 2a. A total of 105 upregulated and 49 downregulated DEMs were identified in WT. A heatmap plot of the related DEMs between WT and normal controls is shown in Fig. 2b. A total of 1894 upregulated and 1715 downregulated DEGs were identified in WT. The DEG distribution between WT and normal controls is presented as a heatmap plot in Fig. 2c.

### CeRNA network construction and functional enrichment analysis

**Fig. 3:**
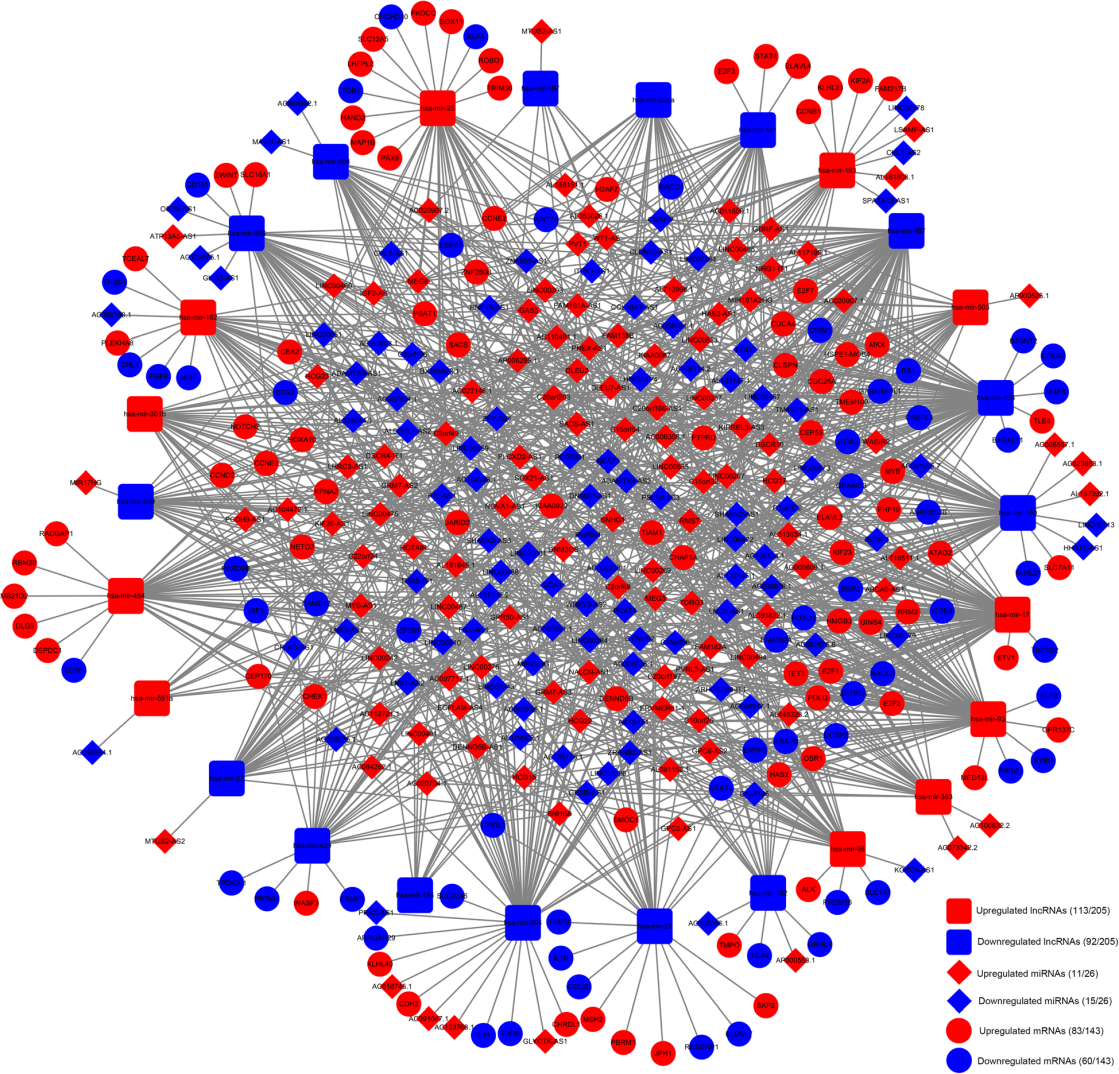
ceRNA regulation network of 205 lncRNAs, 26 miRNAs and 143 mRNAs in Wilms tumour. The red diamonds indicate upregulated lncRNAs, and blue diamonds indicate downregulated lncRNAs; the red triangles indicate upregulated miRNAs, and blue triangles indicate downregulated miRNAs; the red circles indicate upregulated mRNAs, and blue circles indicate downregulated mRNAs. Connectivity between two nodes is expressed as an edge number. ceRNA, Competitive endogenous RNA; lncRNA, long noncoding RNA; miRNA, microRNA; mRNA, messenger RNA

A dysregulated lncRNA-miRNA-mRNA ceRNA network in WT was constructed according to the interactions of 980 DEL-DEM pairs and 235 DEM-DEG pairs between 205 DELs, 26 DEMs, and 143 DEGs. The ultimate lncRNA-miRNA-mRNA ceRNA regulatory network visualized in Cytoscape is presented in Fig. 3.

**Fig. 4:**
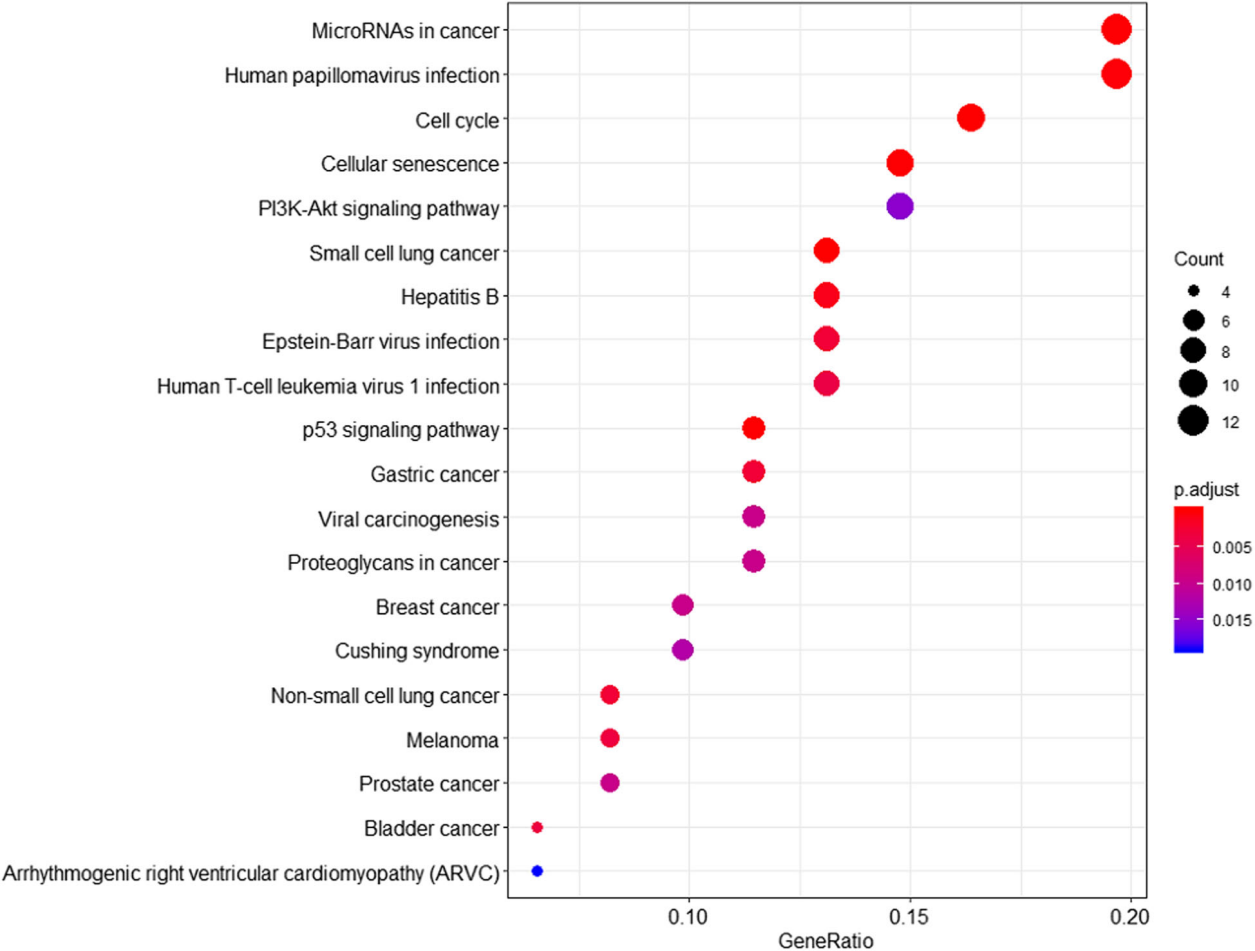
The KEGG pathways enriched in the genes involved in the ceRNA network of Wilms tumour. The X-axis represents the gene ratio for the indicated genes, with the top 20 KEGG pathways shown on the Y-axis. The graph displays only the top 20 KEGG terms. In this graph, the gradient of blue to red denotes a change in the significance of the correlation from low and high, respectively, and the different sizes of the dots represent related mRNA numbers

GO and KEGG analyses were also performed to reveal the functions of the 143 DEGs that were included in the ceRNA network (see Table 1). The DEGs were enriched in 99 “biological processes (BP)” terms, and the top five terms were response to mechanical stimulus, G1/S transition of mitotic cell cycle, cell cycle G1/S phase transition, muscle cell proliferation, and mesenchymal cell differentiation. The DEGs were enriched in 8 “cellular component (CC)” terms, and the top five terms were transcription factor complex, cyclin-dependent protein kinase holoenzyme complex, nuclear chromatin, nuclear transcription factor complex, and Flemming body. The DEGs were enriched in 10 “molecular function (MF)” terms. The top five terms were transcription factor activity (related to RNA polymerase II proximal promoter sequence-specific DNA binding), transcriptional activator activity (related to RNA polymerase II transcription regulatory region sequence-specific DNA binding), proximal promoter sequence-specific DNA binding, transcriptional activator activity (related to RNA polymerase II proximal promoter sequence-specific DNA binding), and transcriptional repressor activity (related to RNA polymerase II transcription regulatory region sequence-specific DNA binding). Additionally, KEGG pathway analysis showed that DEGs were enriched in 30 pathways, such as pathways related to cell cycle, small-cell lung cancer, p53 signalling, microRNAs in cancer, and cellular senescence (Table 1 and Fig. 4).

**Table 1:** The enriched GO terms (top 5) and KEGG pathways (top 5) of the DEGs

Category	Term	ID	Count	FDR
GO BP	response to mechanical stimulus	GO:0009612	11	0.001184756
GO BP	G1/S transition of mitotic cell cycle	GO:0000082	12	0.001333555
GO BP	cell cycle G1/S phase transition	GO:0044843	12	0.001784413
GO BP	muscle cell proliferation	GO:0033002	10	0.002607351
GO BP	mesenchymal cell differentiation	GO:0048762	9	0.009770918
GO CC	transcription factor complex	GO:0005667	10	0.01896361
GO CC	cyclin-dependent protein kinase holoenzyme complex	GO:0000307	4	0.01896361
GO CC	nuclear chromatin	GO:0000790	10	0.01896361
GO CC	nuclear transcription factor complex	GO:0044798	7	0.024102204
GO CC	Flemming body	GO:0090543	3	0.037639391
GO MF	transcription factor activity, RNA polymerase II proximal promoter sequence-specific DNA binding	GO:0000982	15	0.000130468
GO MF	transcriptional activator activity, RNA polymerase II transcription regulatory region sequence-specific DNA binding	GO:0001228	15	0.000130468
GO MF	proximal promoter sequence-specific DNA binding	GO:0000987	14	0.000902732
GO MF	transcriptional activator activity, RNA polymerase II proximal promoter sequence-specific DNA binding	GO:0001077	11	0.000902732
GO MF	transcriptional repressor activity, RNA polymerase II transcription regulatory region sequence-specific DNA binding	GO:0001227	10	0.000902732
KEGG	Cell cycle	hsa04110	10	6.52E-06
KEGG	Small cell lung cancer	hsa05222	8	4.96E-05
KEGG	p53 signaling pathway	hsa04115	7	7.55E-05
KEGG	MicroRNAs in cancer	hsa05206	12	0.00013503
KEGG	Cellular senescence	hsa04218	9	0.000137065

Abbreviations: *FDR,* False discovery rate

### Survival analysis

**Fig. 5:**
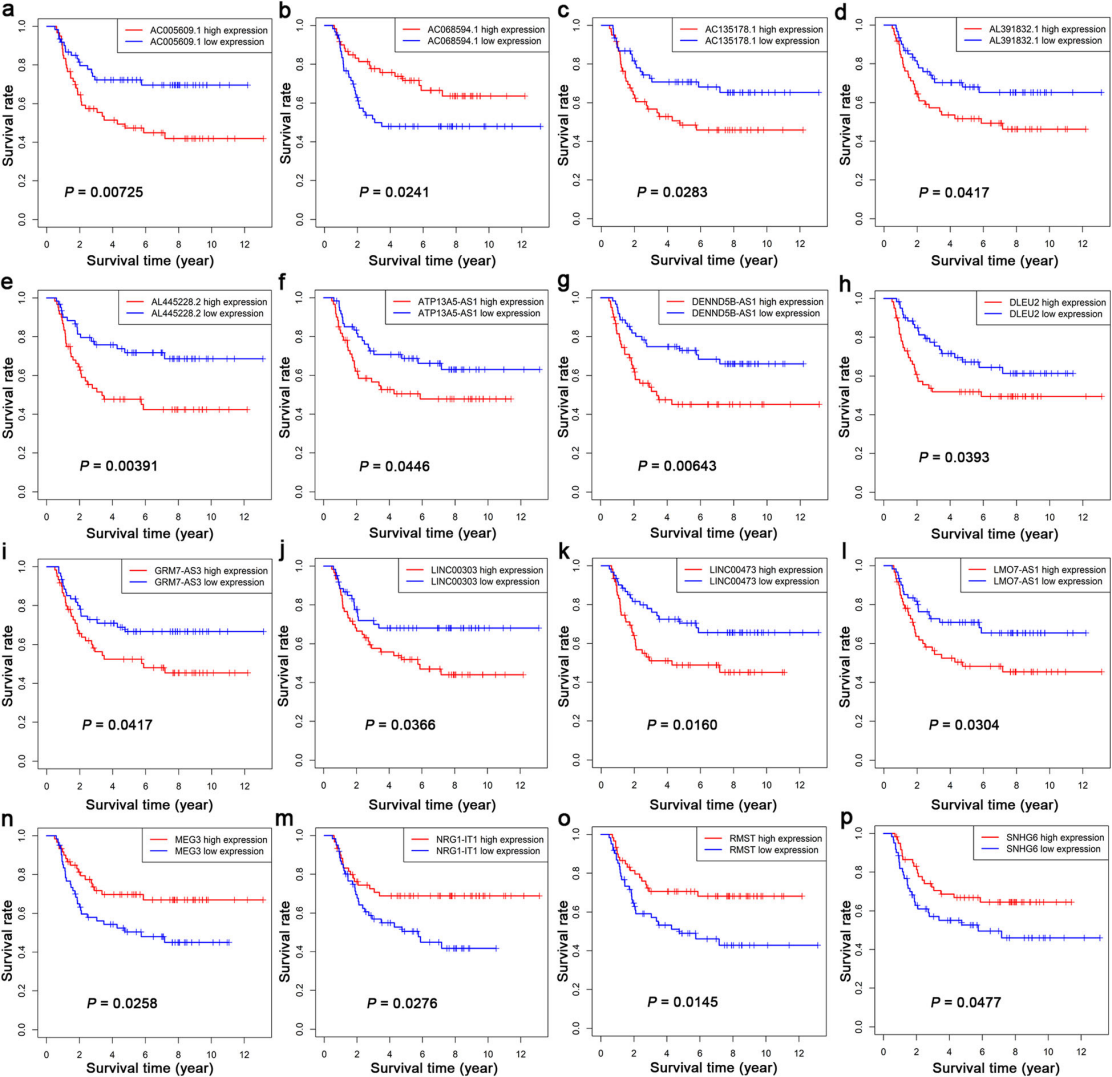
Kaplan-Meier curve of the lncRNAs that are significantly associated with OS in Wilms tumour patients. **a** AC005609.1, (**b**) AC068594.1, (**c**) AC135178.1, (**d**) AL391832.1, (**e**) AL445228.2, (f) ATP13A5-AS1, (**g**) DENND5B-AS1, (h) DLEU2, (**i**) GRM7-AS3, (**j**) LINC00303, (**k**) LINC00473, (**l**) LMO7-AS1, (**n**) MEG3, (**m**) NRG1-IT1, (**o**) RMST and (**p**) SNHG6. The red and blue curves represent samples with higher and lower expression of lncRNAs, respectively

**Fig. 6:**
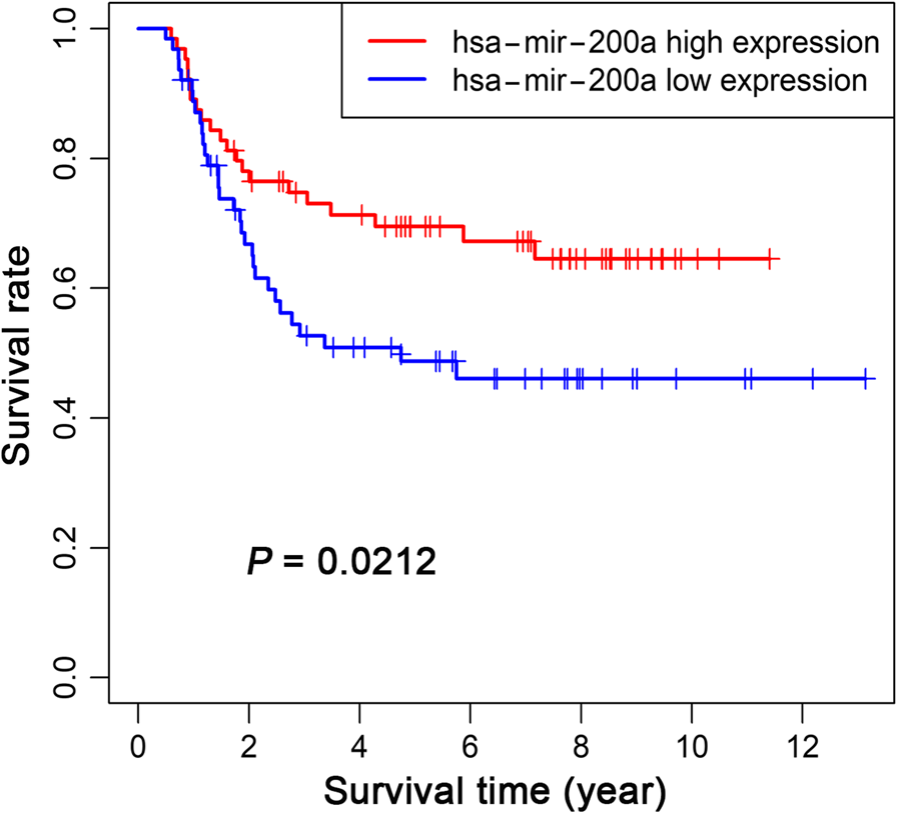
Kaplan-Meier curve of hsa-mir-200a, which was significantly associated with overall survival in Wilms tumour patients. The red and blue curves represent samples with higher and lower expression of miRNAs, respectively

**Fig. 7:**
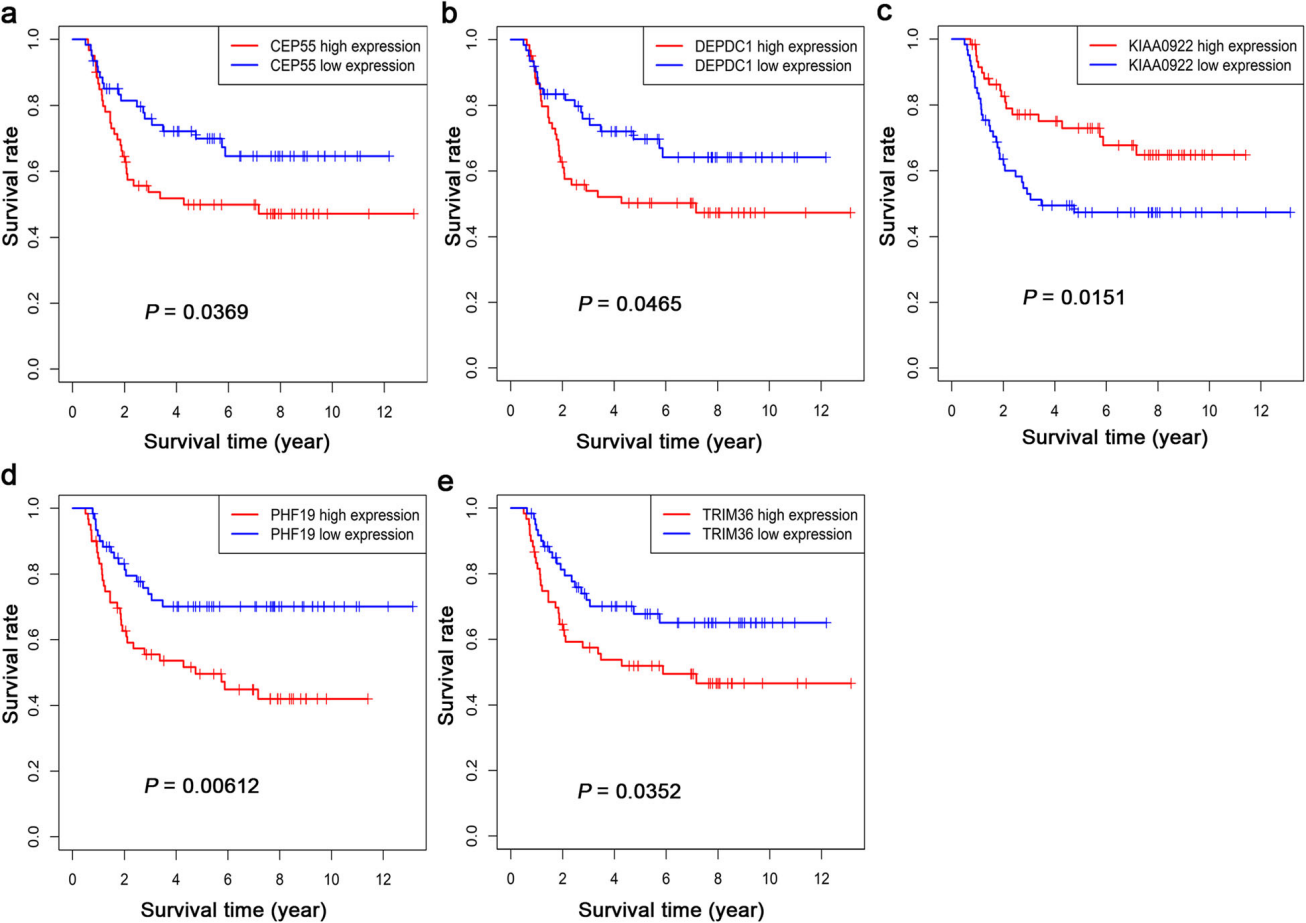
Kaplan-Meier curve of the mRNAs that are significantly associated with OS in Wilms tumour patients. (**a**) CEP55, (b) DEPDC1, (**c**) KIAA0922, (**d**) PHF19 and (**e**) TRIM36. The red and blue curves represent samples with higher and lower expression of mRNAs, respectively

Kaplan-Meier analysis was performed using the combined clinical follow-up information and gene expression profiles of 205 lncRNAs, 26 miRNAs and 143 mRNAs in the ceRNA network in WT samples. According to the analysis, 16 lncRNAs among 205 DELs were closely related to the overall survival of WT patients (Fig. 5). For AC005609.1, AC135178.1, AL391832.1, AL445228.2, ATP13A5-AS1, DENND5B-AS1, DLEU2, GRM7-AS3, LINC00303, LINC00473 and LMO7-AS1, low expression was related to a high overall survival rate in WT patients. High expression of AC068594.1, MEG3, NRG1-IT1, RMST and SNHG6 was related to high survival rates in WT patients. In addition, 1 miRNA among 26 DEMs was closely related to the prognosis of WT patients (Fig. 6). High expression of hsa-mir-200a was related to high survival rates in WT patients. Five mRNAs among 143 DEGs were closely related to the overall survival of WT patients (Fig. 7). For CEP55, DEPDC1, PHF19 and TRIM36, low expression was related to a high overall survival rate in WT patients. High expression of KIAA0922 was significantly related to high survival rates in WT patients.

## Discussion

WT is a kind of renal malignant tumour. Although overall survival in WT patients is constantly improved, disease recurrence and poor prognosis are still the main causes of cancer-related death in childhood [1]. Dysregulated genes are considered to be the major cause of tumorigenesis in WT. Recently, increasing attention has been paid to the crucial roles of the ceRNA network in gene expression regulation at the transcription, post-transcription and translation levels. A previous study reported the regulatory role of ceRNA networks in the proliferation, metastasis and invasion of cancer [15, 16]. To better understand how the ceRNA regulatory network affects WT, large-scale WT data from the TARGET database were analysed, and a dysregulated ceRNA regulatory network in WT was successfully constructed. A dysregulated ceRNA network in WT was constructed according to the interactions of 980 DEL–DEM pairs and 235 DEM–DEG pairs between 205 DELs, 26 DEMs, and 143 DEGs. In addition, Kaplan-Meier curves were generated to identify prognostic biomarkers in WT. Sixteen differentially expressed lncRNAs (AC005609.1, AC068594.1, AC135178.1, AL391832.1, AL445228.2, ATP13A5-AS1, DENND5B-AS1, DLEU2, GRM7-AS3, LINC00303, LINC00473, LMO7-AS1, MEG3, NRG1-IT1, RMST and SNHG6), 1 differentially expressed miRNA (hsa-mir-200a) and 5 differentially expressed mRNAs (CEP55, DEPDC1, KIAA0922, PHF19 and TRIM36) were shown to be significantly associated with the overall survival rate in WT.

Long noncoding RNAs (lncRNAs) are defined as noncoding RNAs longer than 200 nucleotides [17]. lncRNAs were shown to be involved in a variety of biological regulatory functions, including metastasis and tumorigenesis of cancer [18, 19]. A total of 1247 upregulated and 790 downregulated DELs were identified in the present study. A total of 205 DELs were included in the construction of the ceRNA network. Additionally, 16 out of the 205 DELs were associated with overall survival in WT patients (*P* < 0.05). Some differentially expressed lncRNAs in our analysis have been investigated in WT: for example, LINC00473 was show to be capable of decreasing miR-195 expression levels and inhibiting miR-195 function in WT [4]. A dysregulated lncRNA signature including LINC00473/miR-195/IKKα was shown to play a protumorigenic pathogenesis role in WT [4]. Additionally, SNHG6 was shown to be overexpression in WT tissues, the knockout of SNHG6 can inhibit proliferation, migration and invasion of WT cells, which showed that SNHG6 was an oncogene of WT development [20]. The abovementioned molecular experiments partially supported our results in the present study. The identified lncRNAs might serve as prognostic biomarkers and therapeutic targets in WT. In previous studies, RMST was shown to possibly inhibit cell proliferation, invasion, and migration, enhance cell apoptosis, and regulate the cell cycle to act as a tumour suppressor in triple-negative breast cancer [21]. MEG3, a myeloid-related lncRNA, plays a tumour suppressor role in various solid neoplasms [22, 23]. DLEU2 was shown to control miR-16-1 to regulate proliferation, invasion and migration in laryngeal cancer [24]. Few studies, however, have explored the relationship between the abovementioned DELs and tumorigenesis in WT. Additionally, little is known about the regulatory role of AC005609.1, AC068594.1, AC135178.1, AL391832.1, AL445228.2, ATP13A5-AS1, DENND5B-AS1, GRM7-AS3, LINC00303, LMO7-AS1 and NRG1-IT1 in cancer. Therefore, further studies are needed to illuminate the molecular and biological mechanisms of these DELs in WT.

MicroRNAs are single-stranded and 18–25 nucleotide-long noncoding RNAs that target regulated gene expression [25]. DEMs in WT, including 105 upregulated and 49 downregulated DEMs, were summarized in the present study. In the present study, the ceRNA network contained 26 differentially expressed miRNAs. However, only one miRNA (miR-200a) was related to overall survival in WT patients. miR-200a is an important member of the miR-200 family. It has been reported that miR-200a participates in several biological processes to control the progression of cancer [26, 27]. miR-200a was shown to inhibit the survival, proliferation and invasion of glioma cells by target regulating FOXA1[27]. In addition, miR-200a might inactivate BRD4-mediated AR signalling to inhibit the progression of prostate cancer [26]. Moreover, previous studies have reported that miR-200a participates in the development and occurrence of oesophageal cancer, breast cancer and endometrial cancer by target regulating specific genes [28–30]. However, there is no research to clearly elucidate the role of miR-200a in WT. The understanding of the role of miR-200a in the progression of WT is limited and requires more targeted molecular studies.

To further investigate the related cellular mechanisms in WT, GO and KEGG analyses of 143 DEGs in the ceRNA network were performed. The GO analysis results showed that the DEGs were mainly enriched in terms related to response to mechanical stimuli, transcription factor complexes and transcription factor activity (related to RNA polymerase II proximal promoter sequence-specific DNA binding). The KEGG analysis results indicated that DEGs in the ceRNA network were mainly enriched in pathways related to the cell cycle, small-cell lung cancer, p53 signalling, microRNAs in cancer, and cellular senescence. In recent years, many studies have reported the same findings. The PI3K-AKT-p53 signalling pathway is involved in the tumorigenesis of WT and might represent a potential target in the future [31]. MiRNAs, which are single-stranded and 18–25-nucleotide-long noncoding RNAs [25], were involved in regulating proliferation, the cell cycle and apoptosis in WT [32, 33]. Cellular senescence was reported to be responsible for restricted proliferation in WT, and this result was linked to increased p21 expression and was independent of p53 expression [34]. The abovementioned related studies and experiments partially support our GO and KEGG analysis results in the present study.

The major limitation of the present study is that confirmation of the differentially expressed lncRNAs, miRNAs, mRNA and relative pathways in tumour tissues and blood is lacking. Further targeted studies related to this ceRNA network need to be designed to verify and investigate these valuable RNAs in the progression of WT.

## Conclusions

In summary, differentially expressed lncRNAs, mRNAs, and miRNAs were identified, and a functional lncRNA-miRNA-mRNA ceRNA regulatory network for WT tumorigenesis was successfully constructed. Significantly altered lncRNAs, miRNAs and mRNAs might serve as prognostic biomarkers and therapeutic targets for tumorigenesis in WT. The ceRNA regulatory network might illuminate the inner molecular mechanism involved in the progression and tumorigenesis of WT.

## Data Availability

The results published here are in whole or part based upon data generated by the Therapeutically Applicable Research to Generate Effective Treatments (https://ocg.cancer.gov/programs/target) initiative, phs000218. The data used for this analysis are available at https://portal.gdc.cancer.gov/projects/TARGET-WT.

## Abbreviations

ceRNA: competitive endogenous RNA
DEGs: Differentially expressed genes
DELs: Differentially expressed lncRNAs
DEMs: Differentially expressed miRNAs
FDR: False discovery rate
GO: Gene Ontology
KEGG: Kyoto Encyclopedia of Genes and Genomes
lncRNA: Long non-coding RNA
miRNA: microRNA
TARGET: Therapeutically Applicable Research to Generate Effective Treatment
WT: Wilms tumour
